# Risks of medical and developmental delays and associated factors among children from different racial and ethnic groups in Kalamazoo County of Michigan, United States

**DOI:** 10.3389/fped.2026.1634531

**Published:** 2026-05-18

**Authors:** Yvonne Jackson, Oluwasomidoyin Bello, Jessical Saadeh, Tazmi Siddiquey

**Affiliations:** 1 Department of Physical Therapy, Western Michigan University, Kalamazoo, MI, United States; 2 School of Interdisciplinary Health Programs, Western Michigan University, Kalamazoo, MI, United States; 3 College of Medicine, Faculty of Clinical Sciences, University of Ibadan, Nigeria

**Keywords:** developmental delays, developmental risks, low-income, medical delays, medical risks, racial disparities

## Abstract

**Background:**

Medical and developmental risks in early childhood are unevenly distributed across racial and ethnic groups, contributing to persistent health inequities, especially in preschool-aged children. This study assesses the prevalence of medical and developmental risks and delays among children aged 0–5 years from low-income families across different racial groups in Kalamazoo County, Michigan.

**Methods:**

A retrospective cross-sectional study among 10,570 children under five years old, utilizing data from local healthcare and community service providers. Descriptive statistics, chi-square tests, and logistic regression were performed to explore associations between racial and ethnic backgrounds and various outcomes, including medical and developmental risks and delays, with significance set at *p* < 0.05.

**Results:**

The prevalence of medical risk and delay was 54.5% and 7.0% while developmental risk and delay were 13.3% and 6.7%, respectively. Male gender, minority status, younger age, and multiple diagnoses significantly increased the likelihood of both medical and developmental risks. Minority children were 1.3 times more likely to experience medical risks and 1.2 times more likely to experience developmental risks compared to children in the majority group. Black children had 11 times higher odds (AOR = 11.1; 95% CI = 4.8–25.8) and children from the “Other” racial group had 13 times higher odds (AOR = 13.0; 95% CI = 5.9–28.7) of experiencing medical delays compared to White children. Additionally, Black children had higher odds of experiencing developmental delays than White children (AOR = 1.2; 95% CI = 1.0–1.5)

**Conclusion:**

Our study shows that Black children and children from other racial groups are much more likely to encounter medical and developmental challenges compared to White children. These disparities suggest that race, socioeconomic status, and early childhood interventions play a crucial role in influencing medical and developmental outcomes. Addressing these inequities requires targeted policies, such as early screenings, expanded healthcare access, and culturally competent care, to improve health outcomes for minority children in underserved communities. Strengthening early developmental screening, improving continuity of primary care, and guaranteeing timely interventions for high-risk children may further reduce these conditions in this population.

## Introduction

Globally, an estimated 250 million children under five years of age, a critical window for development, were at risk of failing to reach their full developmental potential (1, 2). This worldwide concern is replicated at the national level, with the United States experiencing substantial developmental challenges among its youngest population. In the US, about 1 in 6 children have one or more developmental disabilities or other delays (3). Developmental delay refers to failure to achieve expected milestones in early childhood and can adversely affect social participation later in life (4, 5). Developmental delays affect 10%–15% of preschool-aged children. These delays are often identified during routine clinical observation by primary care providers or when concerns are raised by parents or preschool staff (6).

Life-course research demonstrated that risks in early childhood development with adverse childhood experiences such as poverty, social deprivation, and inequitable access to healthcare have been associated with these risks, highlighting the need to identify and address them as early as during early childhood (7). Early variations in developmental milestone attainment, such as delays in motor or language skills, have been associated with subsequent neurodevelopmental outcomes, underscoring the importance of early identification and intervention to support long-term developmental trajectories (8). National studies have documented racial, ethnic, and socioeconomic disparities in developmental outcomes, with higher prevalence of developmental delays, disabilities, delayed diagnosis, and reduced access to early intervention among children from socially disadvantaged families (9–11). However, existing evidence largely relies on a national survey or parent-reported data and focuses on diagnosed developmental disabilities, limiting the understanding of broader developmental risk and delay patterns during the first five years of life.

Medical risk is the likelihood of an adverse health outcome resulting from environmental or lifestyle exposures, medical conditions, treatment, or procedure (12). Having a medical risk is more prevalent among medically underserved populations, particularly those who face barriers to healthcare access (12–14). Delayed medical care, or medical delay, can contribute to long-term health disparities, impacting both public and individual health outcomes (15). Common reasons for delaying care among children include assumptions that postponing care will not harm health, time constraints, provider scheduling issues, and challenges in covering children's medical expenses and childcare (16).

The prevalence of diagnosed developmental disability in children and adolescents increased from 7% to 9% through 2019–2021 in the US (17). This rate increased significantly, rising from 16% to 18% among boys, older children, non-Hispanic White, Hispanic children, those with private insurance only, and those with less-educated mothers between 2009 and 2017 (10). Additionally, children from low-income families, non-English-speaking households, and specific racial and ethnic minority groups (such as Black and Hispanic people) are exposed to a combination of social, environmental, and economic factors that contribute to uneven risks and make them more likely to experience medical and developmental delays (9, 18, 19).

Primary care providers are central to the early identification of developmental delays through routine developmental surveillance and screening (6). According to the World Health Organization (WHO) and United Nations Children's Fund (UNICEF) report that minority ethnicity and indigeneity can limit access to essential health and support services, particularly prenatal and perinatal care, which may adversely affect child development and increase the risk of developmental disabilities (20). However, comprehensive and timely early childhood interventions—including speech, physical, occupational, and behavioral therapies—can support optimal development and help children reach their full potential (6, 21).

This study is guided by the Social Determinants of Health (SDoH) framework, which emphasizes that health and developmental outcomes are shaped by social, economic, and environmental conditions, including race and ethnicity, socioeconomic status, healthcare access, and neighborhood resources (22). Within this framework, disparities in medical and developmental risks are viewed as consequences of structural inequities that affect early life experiences, healthcare utilization, and access to early identification and support. This perspective provides a rationale for examining racial and ethnic disparities in medical and developmental risks and delays among low-income children.

Although medical and developmental delays in early childhood are well documented, less is known about their prevalence and impact among low-income children, particularly those engaged with pediatric healthcare, wellness visits, public health maternal–child home programs, or early childhood education services. Children with developmental delays often present with medical histories involving multiple risk factors, including maternal and infant biological, psychosocial, economic, and environmental influences (23, 24). However, few studies examine medical risk, medical delay, developmental risk, and developmental delay concurrently within the same population. Most existing research relies on national or state survey data, with limited use of local, system-linked administrative data that reflect real-world service utilization across healthcare and community systems. Moreover, studies integrating clinical diagnoses, emergency department use, and developmental screening data among low-income child populations remain scarce.

This study aimed to assess the prevalence of medical and developmental risks and delays and their associated demographic and healthcare-related factors among children aged 0–5 years from low-income families across racial and ethnic groups in Kalamazoo County. A better understanding of the conditions may support timely diagnosis and early intervention, helping to reduce gaps in healthcare utilization and improve outcomes. We hypothesize that racial and ethnic disparities, along with socioeconomic factors such as age, sex, and minority status, significantly influence the prevalence of medical and developmental risks and delays among children aged 0–5 from low-income families in Kalamazoo County. Furthermore, we examined whether these disparities are influenced by factors like primary care providers, number of ailments diagnosed, and emergency department visits.

## Methods

### Study design, study area, and study population

This study was a retrospective cross-sectional study among preschool-age children in Kalamazoo County, Michigan. Kalamazoo has an average of 3,100 births per year, of which about 50% are low-income. Thus, the annual population of low-income children for 0–5 years was estimated to be 9,300. The study population (*N*) comprised 10,570 children residing in Kalamazoo County, Michigan, who were between the ages of 0 and 5 at any point during the study period and came from low-income families.

### Data collection

The data were collected from local healthcare and community service providers in Kalamazoo County between January 2013 and December 2014. Information about pediatric health care and community services provided by pediatric clinics serving low-income families, hospital emergency departments, public health maternal-child home visitations, and early childhood educational services were included.

Demographic data included race, risk category, individual risk measures, community service visits, healthcare provider(s), community service provider(s), reasons or diagnosis at each visit, and medical and developmental delay. Personal identifiers were used solely to link individuals across system-level datasets, allowing integration of individual-level and visit-level data over time. While the data were collected across multiple facilities using diverse forms and screening tools, a consistent data extraction procedure was applied during dataset integration to ensure consistency for analysis.

### Measures and variables

For this study, low-income status was defined as enrollment in Medicaid, self-pay status indicating lack of insurance, or designation as low-income by the service provider.

Outcome variables: Medical risk, medical delay, developmental risk, and developmental delay were the primary outcome variables. Each outcome was coded as a binary variable “yes” or “no” based on standardized screening and clinical assessment data.

Developmental risks: defined as those identified by the screening process or reported by the health service agency while developmental delay is classified by ICD-9-CM and is identified as medically or developmentally delayed by the health service agency.

Medical risk: defined as the risk of an adverse health outcome from environmental or lifestyle exposure, medical condition, treatment, or procedure, including those falling through medical cracks by risk categories of amount and appropriateness of emergency department usage, immunization, well-child visit status and non-availability of primary care provider while medical delay is those that were late in obtaining medical care.

Number of diagnoses: assessed as primary, secondary, and tertiary diagnosis by the primary care provider at each clinic visit.

Primary predictor: Race and ethnicity were categorized as White and racial and ethnic minority groups, with White children serving as the reference category. Racial and ethnic minority groups included all children identified as non-White. Within the dataset, “Other” included American Indian, Alaskan Native, Asian, Hispanic, Multi-/Biracial, Pacific Islander, and Native Hawaiian/Other Pacific Islander due to small sample sizes.

Covariates: Sex was coded as male or female. Age was treated as a categorical variable (0–2, 3–5, and >5) measured in years. Healthcare utilization variables included primary care visits and emergency department visits, both treated as categorical variables. Diagnostic burden was categorized based on the number of documented medical conditions and included as a categorical variable in the models.

Model specification: All variables included in the multivariable models were selected *a priori* based on existing literature and conceptual relevance to medical and developmental risks/delays in early childhood.

Risk category: Risk was operationalized using multiple indicators, including healthcare utilization, screening practices, and clinical diagnoses. High emergency department utilization was defined as more than three visits, and visit appropriateness was categorized as appropriate or inappropriate. Developmental screening was recorded as a binary variable (Yes/No), based on provider documentation of screening conducted using validated, standardized tools in accordance with protocol. Developmental and medical delays were identified using ICD-9 diagnostic codes, including conditions such as failure to thrive.

### Data analysis

All analyses were conducted using the data obtained from the administrative records with each participant contributing to one observation. Descriptive, Chi-square test, and logistic regression analyses were performed to identify factors associated with the development of these risks and delays among the children using IBM SPSS Version 23.0. Descriptive statistics were used to summarize the prevalence of medical risk, medical delay, developmental risk, and developmental delay. Categorical variables were summarized using frequencies and percentages. Bivariate associations between children characteristics (the age at the end of the study in years), race categorized as White, Black, and others (consisting of seven additional racial and ethnic minority groups), sex, minority group, primary care provider, number of emergency department visits (ED) and medical and developmental outcomes were assessed using chi-square tests. These analyses were conducted to identify unadjusted differences in outcome prevalence across racial/ethnic groups.

Multivariable logistic regression models were then used to examine the independent association between these characteristics and each outcome while adjusting for potential confounders. All outcomes were modeled as binary outcome variables (yes or no). Adjusted odds ratios (AORs) and 95% confidence intervals (CIs) were reported. Race data that were missing or not reported were coded as “Other” to retain these observations in the analytic sample and minimize loss of statistical power. The level of significance is set at *p* < 0.05. Missing data in race was counted as ‘others’, and primary care providers as ‘none’. Race-stratified prevalence results are presented alongside adjusted models to facilitate comparison of unadjusted distributions and adjusted associations (Table 4).

## Results

### Prevalence of medical and developmental risks, medical and developmental delays among the study population

The prevalence of medical risk and delay was 54.5% and 7.0%, with developmental risk and delay at 13.3% and 6.7%, respectively among the assessed 10,570 children under five years old in Kalamazoo County were presented in Table 1.

**Table 1:** Prevalence of medical risk, developmental risk, and medical and developmental delay among the study population.

Variables	Frequency	Percent
Medical risk		
Yes	5,765	55.4
No	4,805	45.6
Medical delay		
Yes	738	7.0
No	9,832	93.0
Developmental risk		
Yes	1,409	13.3
No	9,161	87
Developmental delay		
Yes	705	6.7
No	9,865	93.3

 Table 2 presents the characteristics of children that were significantly associated with medical risk and delay based on bivariate and multivariate analyses. The odds of being at medical risk are higher among children from racial and ethnic minority groups compared to White children (Adjusted Odds Ratio - AOR = 1.3; 95% CI = 1.1–1.5) and children whose primary care provider were “clinic” and “private” (AOR = 1.2; 95% CI = 1.1–1.4) and (AOR = 1.1; 95% CI = 0.9–1.2) respectively compared to children who do not have a primary care provider. In contrast, children aged 3–5 years (AOR = 0.7; 95% CI = 0.6–0.9), children with three or fewer emergency department visits (AOR = 0.04; 95% CI = 0.02–0.07), and those with a pediatric diagnosis (AOR = 0.1; 95% CI = 0.0–0.1) are at lower odds of medical risk.

**Table 2:** Bivariate and multivariate analysis of determinants of medical risks and delays among the study population.

Variables	Medical risk			AOR (95% CI)	Medical delay			AOR (95% CI)
	Yes (%)	No (%)	*χ*2 (*p*-value)		Yes (%)	No (%)	χ2 (*p*-value)	
Age								
0–2	2,526 (0.8)	1,630 (39)	115.9(<0.001)	**1.1** (**0.9–1.3)**	175 (4)	3,981 (96)	94(<0.001)	**0.4** (**0.3–0.6)**
3–5	2,417 (50)	2,467 (51)		**0.7** (**0.6–0.9)**	398 (8)	4,486 (92)		**0.6** (**0.5–0.8)**
>5 (Ref)	822 (54)	708 (47)		-	165 (11)	1,365 (89)		
Sex								
Male	3,055 (56)	2,366 (44)	15	0.9 (0.8–1.1)	382 (7)	5,039 (93)	0.1 (0.789)	1.0 (0.8–1.2)
Female (Ref)	2,710 (53)	2,439 (47)	(<0.001)	-	356 (7)	4,793 (93)		-
Racial and ethnic Minority groups								
Yes	2,285 (51)	2,213 (49)	44	**1.3** (**1.1–1.5)**	252 (6)	4,246 (94)	23 (<0.001)	**0.6** (**0.5–0.7)**
No (Ref)	3,480 (57)	2,592 (43)	(<0.001)		486 (8)	5,586 (92)		
Primary care provider								
Clinic	4,092 (56)	3,238 (44.2)	16	**1.2** (**1.1–1.4)**	0 (0.0)	7,330 (100)	10,570	-
Private	1,299 (52)	1,203 (48.1)	(<0.001)	1.1 (1–1.2)	0 (0.0)	2,502 (100)	(<0.001)	
None (Ref)	374 (51)	364 (49.3)		-	738 (100)	0 (0.0)		
Number of ED visits								
≤3	3,604 (78)	1,035 (22)	300(<0.001)	**0.4** (**0.2–0.4)**	383 (8)	4,256 (92)	43.4(<0.001)	**2.8** (**1.9–3.9)**
>3 (Ref)	1,241 (99)	14 (1.1)		**-**	36 (3)	1,219 (97)		**-**
Number of diagnoses at visits(*n* = 9,053)								
1	790 (49.3)	813 (50.7)	110.1	**1.1** (**0.0–0.1)**	242 (15.1)	1,361 (84.9)	453.0(<0.001)	**1.8** (**1.5–2.2)**
>1 (Ref)	4,722(63.4)	2,728(36.6)	(<0.001)		192(2.6)	7,258(97.4)		

Bold value statistically significant results (confidence interval does not include 1).

The odds of experiencing medical delay are higher in children with three or fewer emergency department visits (AOR = 2.8; 95% CI = 1.9–3.9) and those diagnosed with an ailment (AOR = 1.8; 95% CI = 1.5–2.2). The odds of experiencing medical delay are lower among children from racial and ethnic minority groups compared to White children (AOR = 0.6; 95% CI = 0.5–0.7), children aged 0–2 years (AOR = 0.3; 95% CI = 0.2–0.4) and children aged 3–5 years (AOR = 0.6; 95% CI = 0.5–0.8). Though sex was not significant in the multivariate analysis, an association exists between sex and medical risk. A significant proportion of children with medical risks were male (*p* < 0.001).

 Table 3 reveals the characteristics of children that were significantly associated with developmental risk and delay based on both bivariate and multivariate analyses. The odds of children being developmentally at risk are higher among the racial and ethnic minority group compared to the majority group (White children) (AOR = 1.4; 95% CI = 1.2–1.6). Children diagnosed with one diagnosis have 40% lower odds of developmental risk compared to those with more than one diagnosis (AOR = 0.6; 95% CI = 0.5–0.7). Those with three or fewer emergency department visits have 30% lower odds of developmental risks compared to children with more than three emergency department visits (AOR = 0.7; 95% CI = 0.5–0.8). Regarding age and sex, children aged 0–5 years have two times increased odds of experiencing developmental risk than children older than 5 years old and in, and male children are 1.3 times more prevalent than female children (AOR = 1.3; 95% CI = 1.1–1.5). Meanwhile, the odds of experiencing developmental delay are higher in children aged 0–2 years (AOR = 4; 95% CI  = 2.3–5.1) than in children older than 2 years. Children diagnosed with more than one ailment have 30% lower odds of experiencing developmental delay, while children with three or fewer emergency department visits have 40% lower odds (AOR = 0.7; 95% CI = 0.5–0.8; AOR = 0.6; 95% CI = 0.5–0.8). Additionally, the odds of being developmentally at risk were 3 times higher in children diagnosed at a clinic visit (AOR = 3; 95% CI = 2.0–3.7).

**Table 3:** Bivariate and multivariate analysis of determinants of developmental risks and delays among the study population.

Variables	Developmental risk			AOR (95% CI)	Developmental delay			AOR (95% CI)
	Yes (%)	No (%)	χ2 (*p*-value)		Yes (%)	No (%)	χ2(*p*-value)	
Age								
0–2	537 (13)	3,619 (87)	8 (0.017)	**2 (1.4–2.4)**	449 (11)	3,707 (89)	188(<0.01)	**4** (**2.3–5.1)**
3–5	695 (14)	4,189 (86)		**2 (1.5–2.7)**	195 (4)	4,689 (96)		1.4 (0.9–2.2**)**
>5 (Ref)	177 (12)	1,353 (88)		-	61 (4)	1,469 (96)		-
Sex								
Male	810 (15)	4,611 (85)	25(<0.001)	**1.3 (1.1–1.5)**	383 (7)	5,038 (93)	3	1.1 (0.9–1.3)
Female (Ref)	599 (12)	4,550 (88)		**-**	322 (6)	4,827 (94)	(0.095)	-
Racial and ethnic Minority group								
Yes	664 (15)	3,834 (85)	14(<0.001)	**1.4 (1.2–1.6)**	337 (8)	4,161 (93)	9	**1.2** (**1.0–1.5)**
No (Ref)	745 (12)	5,327 (88)		-	368 (6)	5,704 (94)	(0.004)	-
Primary Care Provider								
Clinic	1,100 (15.0)	6,230 (85.0)	68.0(<0.001)	**3 (2.0–3.7)**	685 (9.3)	6,645 (90.7)	275.5	**8** (**5.0–12.4)**
Private	264 (10.6)	2,238 (89.4)		**2 (1.3–2.5)**	20 (0.8)	2,482 (99.2)	(<0.001)	0.00(-)
None (Ref)	45 (6.1)	693 (93.9)		**-**	0 (0.0)	738 (100.0)		**-**
Number of ED visits								
≤3	507 (10.9)	4,132 (89.1)	27(<0.001)	**0.7 (0.5–0.8)**	287 (6.2)	4,352 (93.8)	23(<0.001)	**0.6** (**0.5–0.8)**
>3 (Ref)	205 (16.3)	1,050 (83.7)		**-**	127 (10.1)	1,128 (89.9)		**-**
Number of diagnoses at visits(*n* = 9,053)								
1	53 (3.3)	156.5 (96.7)	156.60	**0.6 (0.5–0.7)**	15 (0.9)	1,588 (99.1)	127	**0.7** (**0.5–0.8)**
>1 (Ref)	1,103(14.8)	6,347(85.2)	(<0.001)	-	690(9.3)	6,760(90.7)	(<0.001)	-

Bold value statistically significant results (confidence interval does not include 1).

### Analysis of race and medical risks, developmental risks, medical delays, and developmental delays among the study population

Table 4 presents the relationship between racial and ethnic groups and the presence of medical and developmental risks and delays. The odds of being at medical risk were 1.3 times higher in Black children compared to White children (AOR = 1.3; 95% CI = 1.1–1.6). In addition, the odds of experiencing a medical delay were 11 times higher for Black children (AOR = 11.1; 95% CI = 4.8–25.8) and 13 times higher for children in the “Other” racial group (AOR = 13.0; 95% CI = 5.9–28.7) compared to White children. Similarly, Black children and children from the “Other” racial group had higher odds of experiencing developmental risk (AOR = 1.2; 95% CI = 1.0–1.3; AOR = 1.4; 95% CI = 1.1–1.6) compared to White children. Furthermore, Black children have a higher likelihood of having a developmental delay than White children (AOR = 1.2; 95% CI = 1.0–1.5).

**Table 4:** Bivariate and multivariate analysis of risks and delays within the white, black, and other racial groups.

Variable	Medical risk			AOR (95% CI)	Medical delay			AOR (95% CI)
	Yes (%)	No (%)	χ2 (*p*-value)		Yes (%)	No (%)	χ2 (*p*-value)	
Race			108 < 0.001)				20(<0.001)	
Others	542 (41.8)	756 (58.2)		1.2 (0.9–1.5)	90 (6.9)	1,208 (93.1)		**13** (**5.9–28.7)**
Black	1,745 (54.1)	1,481 (45.9)		**1.3 (1.1–1.6)**	173 (5.4)	3,053 (94.6)		**11** (**4.8–25.8)**
White (Ref)	3,478 (57.5)	2,568 (42.5)		-	475 (7.9)	5,571 (92.1)		-

Variable	Developmental risk			AOR (95% CI)	Developmental delay			AOR (95% CI)
	Yes (%)	No (%)	χ2 (*p*-value)		Yes (%)	No (%)	χ2 (*p*-value)	
Race			16(<0.001)				8 (0.016)	
Others	207 (15.9)	1,091 (84.1)		**1.4 (1.1–1.6)**	91 (7.0)	1,207 (93.0)		1.1 (0.9–1.4)
Black	459 (14.2)	2,767 (85.8)		**1.2 (1.0–1.3)**	246 (7.6)	2,980 (92.4)		**1.2** (**1.0–1.5)**
White (Ref)	743 (12.3)	5,303 (87.7)		-	368(6.1)	5,678(93.9)		-

Bold value statistically significant results (confidence interval does not include 1).

## Discussion

This study examined the prevalence of medical and developmental risks and delays among children under five years of age across racial groups in Kalamazoo County and identified associated risk factors. Early identification of developmental risks and delays is critical, as it allows for timely intervention services that can significantly enhance children's functional skills and outcomes (25). Guided by the Social Determinants of Health (SDoH) framework and a life-course perspective, our findings are interpreted within the framework of social, economic, and structural contexts that shape early childhood health trajectories. This approach emphasizes that medical and developmental outcomes are influenced not only by biological factors, but also by inequities in access to care, timing of diagnosis, cumulative disadvantage exposure during development period (7, 22, 26).

The prevalence of developmental risk and delay among the preschool children studied was 13.3% and 6.7%, respectively. This is consistent with national estimates of 10%–15%, with only about one-fifth of these children receiving yearly intervention before the age of three (27). However, a higher prevalence rate was reported among low-income populations nationally (21%–42%) and in Michigan (25%) (28). The discrepancy in prevalence rates likely reflect variation in study populations, settings, and use of single-county data.

For medical risk, our study found a prevalence of 55%, with 7.0% experiencing delays in medical care. In contrast, Gertz et al. reported a 17% rate of delayed medical care during the COVID-19 pandemic (15). The higher rate in their study may be due to pandemic-related factors, which may have contributed to increased delays in care. It is also important to note that there was a pre-existing downward trend in children's healthcare access and utilization from 2016 to 2019, which further deteriorated during and after the pandemic, contributing to increased risks and potential long-term adverse outcomes (27).

Race was significantly associated with both medical and developmental risks and delays, with Black children and those in the “Other” racial category experiencing higher risk compared to White children. These findings align with national data demonstrating racial disparities in developmental outcomes (11, 17, 29). The National Health Interview Survey similarly indicated that Black children were more frequently diagnosed with intellectual disabilities compared to other racial groups (10). In Kalamazoo County, a study reported that Black infants face the poorest health outcomes, a disparity that persists across all socioeconomic levels, underscoring the ongoing impact of race on health disparities (30). Although some studies suggest socioeconomic status may outweigh race in certain contexts. For instance, Bitsko and colleagues found that in children with attention-deficit/ hyperactivity disorder diagnosis by race and ethnicity, there were higher estimates among non-Hispanic White and non-Hispanic Black children compared with non-Hispanic Asian and Hispanic especially children living in lower-income households, children with public health insurance, and children living in rural areas (31). Another study reported that developmental disabilities were more prevalent among non-Hispanic White children, those with public insurance, children whose mothers had less than a college education, and children living below 200% of the federal poverty line (17). These findings indicate that socioeconomic status can substantially influence health outcomes independent of race. However, children from racial and ethnic minority groups continued to exhibit higher rates of developmental risks and delays (17, 32).

In addition, younger age, male gender, minority status, and multiple diagnoses were associated with higher odds of medical and developmental risks and delays, consistent with prior research (9, 17, 20, 33). Increased healthcare visits, particularly more than three emergency department visits, was also associated with higher likelihood of experiencing medical and developmental risks and developmental delay, likely reflecting greater medical complexity and delayed identification. This is consistent with the findings of Russell et al. (34). Increased healthcare visits are often related to the diagnosis and management of developmental delays, and children with multiple diagnoses are likely to experience developmental delays. Delayed identification and intervention may be associated with a greater risk of more severe impairments, highlighting the need for timely diagnosis and intervention.

In our study, primary care providers assessed all children for medical and developmental risks and delays. A previous study examining children's healthcare utilization and community engagement reported a 16% prevalence of developmental delay was associated with limited physician visits (34). These findings underscore the central role of primary care providers in identifying medical and developmental risks through routine surveillance and screening, thereby enabling timely interventions to prevent or mitigate long-term developmental problems (6, 34). Overall, consistent access to primary care is crucial for supporting early detection, intervention, and improved developmental outcomes.

### Limitations and strengths

Due to the secondary and retrospective nature of the data, this study is subject to several limitations. Causal relationships between medical and developmental risks and delays cannot be established, and the findings may not be generalizable beyond the population and geographic context in which the data were collected. The aggregation of racial and ethnic categories may have obscured heterogeneity within the “Other” category; as such, findings related to this group should be interpreted with caution. Future research using larger and more diverse samples may allow for disaggregated analyses to better characterize within-group variation. The results presented reflect patterns from a pre-pandemic healthcare context and may not capture changes in healthcare access or service utilization that occurred during or after the COVID-19 pandemic. Another limitation is the use of administrative data collected across multiple clinics using different forms and screening tools, which may have introduced measurement variability; however, datasets were linked and standardized during integration to ensure consistency. Despite these limitations, the large sample size increased statistical power and enabled the examination of interaction effects. While the findings should be interpreted conservatively, the study provides descriptive insight into patterns of medical and developmental delay among children under five years of age in Kalamazoo and includes data from children across diverse racial and ethnic backgrounds.

### Recommendations

Based on our findings, we recommend expanding routine developmental screening in primary care and community settings to support early identification of at-risk children. In Kalamazoo County, efforts should focus on improving continuity of care and reducing barriers to accessing primary care. Targeted interventions should be made available for high-risk populations, particularly racial and ethnic minority groups and low-income families. Integrating healthcare, public health, and early childhood education systems can strengthen early detection and management of developmental concerns. Additionally, policies addressing SDoH such as poverty, caregiver education, and access to community resources are essential for improving long-term outcomes. Future cohort studies are needed to better understand causal pathways, assess the effectiveness of interventions, and examine longitudinal outcomes linking early medical and developmental risks to long-term child health and developmental trajectories.

## Conclusion

This study observed higher prevalence of medical and developmental risks and delays among children from racial and ethnic minority groups, with stronger associations noted among male children, those with multiple diagnoses, and those with more than three emergency department visits. While early intervention services were not directly measured, these findings underscore the potential importance of early identification of children who may be at elevated risk for medical and developmental delays, particularly among racial and ethnic minority children, males, and children aged 0–5 years. Addressing disparities in healthcare access may help reduce the burden of medical and developmental risks.

Although causal effects cannot be inferred from this study, existing evidence suggests that early intervention services for children at risk of developmental delays may be beneficial. Such services may include referrals to physical therapy and other supportive interventions, which have been associated with improvements in motor development and functional outcomes. In addition, the integration of occupational and speech therapy within developmental services has been associated with gains in daily functioning, communication, and social skills. Developmental services that provide tailored support may help address specific developmental needs and support broader cognitive, emotional, and social development.

## Data Availability

The raw data supporting the conclusions of this article will be made available by the authors, without undue reservation.
